# The Role of Dietary Fats in the Development and Treatment of Endometriosis

**DOI:** 10.3390/life13030654

**Published:** 2023-02-27

**Authors:** Angelika Marcinkowska, Magdalena Górnicka

**Affiliations:** Department of Human Nutrition, Institute of Human Nutrition Sciences, Warsaw University of Life Sciences, Nowoursynowska St. 159C, 02-776 Warsaw, Poland

**Keywords:** dietary fats, endometriosis, inflammation, pelvic pain, women

## Abstract

Endometriosis is an estrogen-dependent disease in women of childbearing age that affects approximately 5–15% of the female population. The etiology of endometriosis is complex, multifaceted, and not fully understood. In endometriosis, which is an estrogen-related chronic inflammatory condition, estrogen plays a major role in endometrial cellular growth. High estrogen levels could be another risk factor for developing endometriosis. The aim of this review is to update knowledge on the impact of dietary fats on the development of endometriosis and chronic inflammation in women with endometriosis and diet therapy. Dietary fat may be linked with the progression and development of endometriosis, but studies have been contradictory due to various issues including sample size, different study designs, and different methodological aspects. Results have shown that the risk of endometriosis may increase with a higher consumption of products rich in saturated fats, especially palmitic acid and trans-unsaturated fatty acids. Monounsaturated fats and omega-3 polyunsaturated fatty acids may likely be connected with a lower risk of developing endometriosis and with reductions in the severity of disease. Monounsaturated fats, omega-3 polyunsaturated fatty acids, and a suitable eicosapentaenoic acid to arachidonic acid ratio can be used in diet therapy to improve quality of life by reducing pain and inflammation. Further research is needed in order to fully understand the influence of dietary fats on the risk of development of this disease.

## 1. Introduction

Endometriosis is an estrogen-dependent disease in women of reproductive age, with features of chronic inflammation [1]. It is defined by the presence of endometriotic cell clusters beyond the uterine cavity, which can be found among others in the pelvic cavity, fallopian tubes, ovaries, upper abdomen, sigmoid colon, and the appendix [2]. The primary symptoms of endometriosis are chronic pain in the abdomen, dysmenorrhea [3], and deep dyspareunia [4]. Furthermore, endometriosis may also reduce fertility [5]. This disease’s prevalence affects approximately 5–15% of women of childbearing age [6], and 30–50% experience infertility [7]. Four pathological stages of endometriosis have been described, namely I—minimal, II—mild, III—moderate, and IV—severe, taking into account the presence and location of adhesions and the depth and location of implants [8]. According to the latest ESHRE guidelines (2022), laparoscopy with biopsy, previously considered the gold standard method for diagnosing endometriosis, is not necessarily the best and required method for an accurate diagnosis today [9]. Endometriosis is treated pharmacologically and surgically, but also increased attention is being dedicated to supplementary treatment [10].

The etiology of endometriosis is complex, multifaceted, and not fully understood [11]. Genetic profile, retrograde menstruation, inflammation, hormonal activity, immune dysfunction, organochloride burden, oxidative stress, metaplastic processes, and apoptosis suppression have been mentioned as risk factors [12]. In addition, an imbalance between the adhesive, invasive, and proliferative properties of endometrial cells was found, as well as an increase in the production of the pro-inflammatory factors responsible for the development of endometriosis. The review results also indicate that endometriosis resembles an immune-dependent disease with the breakdown of immunosuppressive mechanisms and autoimmune background [13]. Garitazelaia et al. suggested that endometriosis usually appears together with a few other phenotypes. These involve a list of autoimmune diseases, anthropometric traits related to leanness in adulthood, and female reproductive traits, including altered hormone levels and those linked with prolonged exposure to menstruation [14].

In endometriosis, estrogen plays a major role in endometrial cellular growth [15]. Estrogen can promote, migrate, survive, proliferate, and adhere to epithelial cells and endometrial stroma [16]. It is produced through the ovaries, fatty tissue, and skin and might be produced locally by a positive feedback loop among estrogen, aromatase, prostaglandin E2 (PGE2), and cyclooxygenase-2 (COX-2) [17]. High estrogen levels could be another risk factor for developing endometriosis, an estrogen-dependent disease. The connection between diet and the occurrence of estrogen-dependent diseases, for example, endometrial or breast cancer, has been demonstrated. Nutrition and nutritional status may play a role in the etiology of the disease by affecting blood estrogen levels [18]. Additionally, dietary factors may be related to symptoms or physiologic processes believed to be associated with endometriosis due to their role in regulating inflammation, prostaglandin, steroid hormone metabolism, and menstrual cycle reducing oxidative stress [19]. Huang et al. demonstrated that dietary factors could influence serum sex-hormone activity and concentrations [20]. Some dietary fats could modify endogenous hormone metabolism. Intake of fatty acids has been related to increased endogenous estrogen levels [21].

Endometriosis is related to an inflammatory response, leading to endothelial dysfunction [22]; thus, inflammation plays a key role in the pathogenesis of endometriosis. The inflammatory response can promote the generation of chemokines and cytokines in the peritoneal cavity [23]. The inflammatory mediators in endometriosis include prostaglandins, interleukins (IL), vascular endothelial growth factor (VEGF), tumor necrosis factor (TNF-α), and nerve growth factor (NGF) [24,25]. Inflammatory response stimulates the secretion of proinflammatory neuromediators such as neuropeptide Y (NY) and NGF [26], promoting neuroagiogenesis [27].

Scholl et al. showed that TNF-α might be a key cytokine involved in the inflammatory aspect of this disease [28]. Women with endometriosis had increased TNF-α in tissues [29]. Furthermore, IL-16 existing in the peritoneal fluid may be involved in the pathogenesis of endometriosis by initiating or sustaining the inflammatory response in the peritoneal cavity [30]. Neutrophils and macrophages are inflammatory cells involved in inflammation [31]. Macrophages infiltrating the ectopic lesions express typical markers of alternative activation, favoring the growth of the lesions and promoting their angiogenesis. The nuclear factor-ΚB-dependent (NF-ΚB) pathway is also engaged, with the transactivation of responsive gene elements controlling angiogenesis and tissue remodeling [32]. In addition, estrogens may regulate macrophages [33], suggesting a relationship between estrogens and the immune reaction in endometriosis [22].

In addition, dietary fats can affect inflammatory responses [34]. Dietary fat is an essential component of the human diet, which is a source of energy and nutrients, and bioactive fatty acids can influence cell metabolism [35]. The difference depends on the fatty acid composition. In vitro studies on the survival of endometrial cells from women with and without endometriosis have reported that endometrial cells are influenced by the fatty acid content of the culture media [36]. Specific dietary fatty acids have been proven to influence the circulating levels of inflammatory markers such as Interleukin-6 (IL-6) and others, which are found in higher levels among women with endometriosis [37]. Bedaiwy et al. found that the inflammatory markers increased with specific fatty acid exposure [38]. In contrast, the application of a high-antioxidant diet (HAD) reduced oxidative stress and the progression of endometriosis development [39]. In addition, certain mediators, such as leukotriene B4 (LTB4) and PGE2, are believed to be associated with pelvic pain in endometriosis [40]. Arachidonic acid (AA) is the most important component of Omega-6 fatty acid (n-6) PUFA, which plays a substrate role in producing mediators such as LTB4 and PGE2. Conversely, eicosapentaenoic acid (EPA), a significant component of Omega-3 fatty acid (n-3) PUFA, plays a role in the biosynthesis of leukotriene B5 (LTB5) and prostaglandin E3 (PGE3), which have less of an inflammatory effect compared with PGE2 and LTB4 [41]. EPA can inhibit the conversion of AA to PGE2 and LTB4 [40].

Therefore, this review aims to update knowledge on the impact of dietary fats on the development of endometriosis and chronic inflammation in women with endometriosis and diet therapy.

## 2. Literature Search

The present review summarizes the findings on the association between dietary fat and diet therapy and the risk of endometriosis among adult women. The literature search was conducted between October 2022 and January 2023 in PubMed using specific keywords: “dietary fat,” “saturated fat,” “monounsaturated fat,” “polyunsaturated fat,” “saturated fat,” and “trans-fat” in combination with “endometriosis” “endometriosis diet,” and “endometriosis risk.” Original articles on the influence of dietary fats on the risk of developing and the occurrence of endometriosis published in English were used. The review contains animal trials and in vitro studies, clinical trials, controlled trials, cohort studies, randomized controlled trials, systematic reviews, and meta-analyses.

## 3. Total Fats (TF)

A high-fat diet is associated with different health effects, both positive and negative. The WHO recommends reducing total fat intake to less than 30% of total energy to help prevent diet-related and metabolic diseases in the adult population [42]. EFSA recommends a total fat intake of 20–35% energy [43], similar to Polish recommendations [44]. 

There is compelling data indicating that high-fat meals promote the translocation of endotoxin (e.g., lipopolysaccharide (LPS)) into the bloodstream, stimulating innate immune cells, leading to a transient postprandial inflammatory response [34]. High fat intake can increase oxidative stress and inflammation, which are the major features of endometriosis [38]. According to a meta-analysis, the results of the studies showed no significant association between TF intake and the risk of endometriosis [45]. A prospective study (Nurses’ Health Study II) found that TF consumption was not associated with endometriosis risk [19]. Three case-control studies investigated the effect of TF consumption on the risk of endometriosis, but their results were inconclusive. Trabert et al. observed that a higher intake of TF was associated with a reduced risk of endometriosis [46]. Samaneh et al. showed that TF consumption was not associated with endometriosis risk [47]. However, a study that examined the relationship between benign ovarian tumors (BOTs) and nutrients, primarily dietary fat, found that the risk of benign ovarian tumors (BOTs) was elevated for the highest vs. the lowest quartile of TF intake [48]. 

The results of studies on animal models showed that feeding mice a high-fat diet containing 72% of total energy over 4 weeks significantly increased circulating endotoxin concentrations compared to mice fed a low-fat control diet. In addition, the study observed elevated inflammatory biomarkers such as TNF-α, IL-1, and IL-6 in the liver, adipose tissue, and muscles. The effects of a high-fat diet may be due to the activation of the inflammatory signaling pathways [49]. In other animal studies, a high-fat diet (more than 45% energy) has been found to affect endometriosis progression. Furthermore, this study confirmed the association between a high-fat diet and increased oxidative stress and inflammation [50]. 

The study results currently available do not provide clear recommendations regarding total fat intake for women with endometriosis. The association between dietary fat intake and the risk of developing endometriosis is unclear (Table 1). Further research is needed to fully understand the influence of total fat intake quantity on the risk of developing this disease.

## 4. Saturated Fatty Acids (SFAs)

SFAs contain only single bonds and include, i.a., myristic acid (MA, 14:0), palmitic acid (PA, 16:0), and stearic acid (SA, 18:0). Saturated fatty acids dominate in animal products such as butter, lard, red meat, cheese, and milk, and in some plant fats, such as palm oil, palm kernel oil, and coconut oil [52]. Palmitate is the main component of palm oil and can also occur in dairy products and meat [53]. It is the key component of highly processed foods, often used in the food industry. SFAs have been associated with adverse effects on health [54]. 

Dietary guidelines recommend limiting the consumption of SFAs to help prevent diet-related and metabolic diseases. The WHO recommends that SFA consumption should account for less than 10% of the daily energy intake of the adult population [42]. The EFSA and the Polish Dietary Reference Intakes recommend an intake of SFAs at the lowest possible level in a diet that provides adequate nutritional value in an adult [43,44]. 

SFAs are an essential structural part of bacterial endotoxins [55] and activate the proinflammatory LPS. Some cells of the innate immune system and macrophages have toll-like receptor (TLR) 4 that recognizes LPS [56]. LPS-mediated signaling through TLR4 induces the activation of NF-κB, a transcription factor, which subsequently turns on the expression of TNF-α, IL-1, IL-6, and IL-8 proinflammatory cytokines [57]. Lee et al. demonstrated that SFAs could directly stimulate inflammatory gene expression through TLR4 signaling in vitro [58]. SFAs induce inflammation partly by mimicking the actions of LPS. Palmitic acid (PA) has been shown to induce the activation of TLR4 receptors in hypothalamic microglia and to stimulate cytokine release [59]. PA may also increase endometriosis risk by producing endogenous estrogen [19]. Increased estrogen levels may induce inflammation in endometriosis by stimulating certain prostaglandins [60]. 

The results of a meta-analysis indicate a significant relationship between SFA intake and the risk of endometriosis [45]. Although a prospective cohort study from the Nurses’ Health Study II reported that SFA consumption was not associated with endometriosis risk, PA intake was significantly associated with an increased risk of endometriosis [19]. A population-based case-control study found inverse associations between SFA consumption and endometriosis risk [46]. Conversely, a case-control study demonstrated that the risk of benign ovarian tumors (BOTs) was elevated for the highest vs. the lowest quartile of saturated fat consumption [48]. A further case-control study found that the intake of SFAs by women in the endometriosis group was similar to that of women in the control group without endometriosis [51]. 

In conclusion, the relationship between SFAs and the risk of developing endometriosis is unclear (Table 1); however, taking into account the results, especially the meta-analysis, women with endometriosis should be recommended a diet low in SFAs and, in particular, limit the intake of PA, to which animal products are a major contributor.

## 5. Monounsaturated Fatty Acids (MUFAs)

MUFAs are chemically classified as fatty acids containing a single, double bond [61], primarily derived from rapeseed oil, olive oil, nuts, and whole milk products [52]. The most common MUFAs in the diet are oleic acid (OA, 18:1 *n*-9), followed by palmitoleic acid (PO, 16:1 *n*-7), and vaccenic acid (VA, 18:1 *n*-7) [61]. The WHO and the Polish Dietary Reference Intakes have not provided dietary recommendations for MUFA intake [42,43,44].

MUFAs reduce the generation of inflammatory and reactive oxygen species [22]. Oleic acid is known for its anti-inflammatory effects [62]. Oleic acid supplementation increases anti-inflammatory cytokine levels (e.g., IL-10) and decreases proinflammatory cytokine levels such as IL-1β and TNF-α in septic mice [63]. In addition, palmitoleic acid is also demonstrated to have anti-inflammatory properties. An in vitro study reported that palmitoleic acid reduces cytokine and adhesion molecule production and downregulates pro-inflammatory genes such as COX-2, IL-6, NFkB, and monocyte chemoattractant protein-1 (MCP-1), and also increases the abundance of vaccenic acid, which is an anti-inflammatory mediator [64].

A meta-analysis found no association between MUFA intake and endometriosis risk [45]. Similarly, a prospective study in the Nurses’ Health Study II demonstrated that MUFA intake was not associated with endometriosis risk [19]. Conversely, a population-based case-control study found inverse associations between MUFA consumption and endometriosis risk [46]. Similarly, a case-control study demonstrated that a higher intake of MUFAs and OA was associated with a lower risk of endometriosis [47]. Another case-control study showed that women in the highest quartiles of monounsaturated fat consumption were at higher risk of developing an endometrial tumor; however, the risk was not significant after the adjustment for polyunsaturated fat consumption [48]. Currently, available research results (Table 1) do not provide strong evidence of a link between MUFA consumption and endometriosis.

However, the recommendation to increase MUFA–rich foods intake seems to be supported by studies showing that women with endometriosis consume less MUFAs than those without the disease [65]. Additionally, the Mediterranean diet, characterized by a high intake of olive oil, a good source of MUFAs, has been linked to lower levels of inflammation. More research is needed to provide strong evidence of the importance of MUFAs in the dietary treatment of endometriosis.

## 6. Polyunsaturated Fatty Acids (PUFAs)

PUFAs contain a minimum of two double bonds [66]. They have been classified as n-6 or n-3 fatty acids, depending on the location of the first double bond and the methyl group. Linoleic acid (LA, 18: 2n-6), the precursor of n-6 PUFA, is an essential fatty acid that cannot be synthesized by mammals and is predominant in vegetable oils, nuts, and seeds. LA is metabolized into AA (20:4 n-6). α-Linolenic acid (ALA, 18:3 n-3) belongs to n-3 PUFAs. ALA is also an essential fatty acid that cannot be synthesized in humans and must therefore be obtained by diet. Products rich in ALA are leafy vegetables, nuts, soybeans, flaxseed, chia, and vegetable oils. ALA is metabolized into eicosapentaenoic acid (EPA, 20:5 n-3) and, finally, to docosahexaenoic acid (DHA, 22: 6n-3) [67]. Furthermore, EPA and DHA may be obtained from fish, fish oil supplements, and other marine products [68]. The WHO dietary guidelines recommend replacing saturated and trans-fats with unsaturated fats—particularly polyunsaturated fats. EFSA and the Polish Dietary Reference Intakes recommended a minimum intake of 4% of energy LA and 0.5% energy ALA, respectively. In addition, 250 mg of EPA and DHA is recommended for adults [42,43,44]. The recommendations above help to prevent diet-related and metabolic diseases.

Many studies show a strong association between dietary PUFAs intake and reduced inflammation [69], which, as indicated above, plays a vital role in the pathogenesis of endometriosis [70]. PUFAs n-3, especially EPA and DHA, play a major role in immunological responses. They suppress the genes involved in inflammation and alter the cell membrane composition by displacing n-6 PUFA and cholesterol [71]. PUFA n-3 has been proven to play a role in regulating and reducing inflammatory prostaglandins and cytokines (IL- 1, 2, and 6, TNF-α) [72]. Consequently, n-3 PUFAs, such as EPA and DHA, may reduce inflammation [73]. Tomio et al. found that EPA-derived metabolites, generated through the 12/15 lipoxygenase pathway, inhibited endometriotic lesions in a mouse model [74]. Furthermore, 12/15-lipoxygenases may act as coactivators of PPAR-γ [75]. Prostaglandin levels may be a pathogenic factor influencing both dysmenorrhea and endometriosis. Dietary n-6 PUFAs are the precursors of the proinflammatory prostaglandins PGE2 and PGF2α, which likely increase uterine cramps and cause the painful symptoms of endometriosis; however, prostaglandins PGE3 and PGE3α, derived from the n-3 fatty acids, have been associated with reduced inflammation and less pain [76].

Currently, available study results do not provide explicit recommendations regarding the total intake of PUFA, EPA+DHA, or LA and ALA for women with endometriosis. Increasing PUFA n-3 intake seems justified, given that PUFA n-3 reduces the production of pro-inflammatory eicosanoids by competing with the n-6 PUFA, repulsing AA in membrane phospholipids, and reducing pain. However, in a meta-analysis, Arab et al. reported no significant association between the intake of PUFAs and the risk of endometriosis [45]. A comprehensive review found that increased consumption of fish oil containing high amounts of n-3 reduced pain intensity and led to less use of painkillers and a shorter duration of pain in women with endometriosis [77]. A double-blind, randomized, placebo-controlled trial that examined the relationship between nutritional supplementation with 1000 mg fish oil and modification of endometriosis symptoms in adolescent girls and young women with endometriosis found that supplementation resulted in pain reduction [78]. In addition, the cohort study results showed a significant reduction of the symptoms in endometriosis patients after 3 months of treatment with various n-3/6 PUFA supplements compared to the control groups. In addition, a significant reduction in inflammatory markers such as PGE2 and carcinoma antigen 125 (CA-125) was found [79]. A prospective cohort study within the Nurses’ Health Study II found that a diet rich in n-3 fatty acids decreased the risk of endometriosis. Women with the highest consumption of n-3 fatty acids were 22% less likely to be diagnosed with endometriosis than women with the lowest intake [19]. Results of the case-control study showed that healthy women (control group) had a higher intake of n-3 and n-6 PUFAs than women diagnosed with endometriosis, which was most likely a relevant factor in the observed decreased risk of developing endometriosis [51]. Similarly, other case-control studies demonstrated that the consumption of EPA and DHA was associated with decreased endometriosis risk [47]. Additionally, Deutch et al. showed that higher consumption of marine n-3 correlated with lower categorized and reduced menstrual symptoms [80], which are troublesome symptoms in endometriosis. Hopeman et al. found that women with high serum EPA levels had an 82% lower risk of endometriosis than women with lower serum EPA levels [66]. Conversely, some research results have indicated that a higher consumption of PUFAs is associated with endometrioid, serous, and teratoma tumors [48] and found no correlation between endometriosis risk and serum phospholipid levels (n-6, n-3, PUFA, MUFA). However, the ratio of EPA to AA was a significant factor in the disease severity [81].

Some animal model studies have also investigated the link between PUFAs and endometriosis. Increased exposure to the EPA has been proven to significantly suppress the in vitro endometrial cell survival compared to those cultured in media low in long-chain n-3 fatty acids or low or normal n-3:n-6 PUFA ratios. Furthermore, the survival of endometrial cells was not affected when cultured in a medium with high n-6 AA content [36]. In addition, in a female rabbit model of surgically induced endometriosis, ALA n-3 PUFAs decreased concentrations of PGE2 and prostaglandin F2α (PGF2α) and endometrial implant diameter [82]. Moreover, a prospective, single-blind, randomized, controlled experimental study in a rat model observed that n-3 PUFAs caused a considerable regression of endometriotic implants [83]. In addition, supplementation with fish oil reduced the development of spontaneous endometriosis-associated adhesions [84]. A prospective, randomized experimental study on an endometriosis rat model investigated the anti-inflammatory effect of n-3 EPA compared with n-6 LA. The findings of the study demonstrated that EPA supplementation might be a valid strategy for treating endometriosis [41]. In animal models, the anti-inflammatory impact of n-3 PUFAs is evaluated as a potential treatment target in the early establishment of endometriosis [85]. The controlled experimental study of rat models conducted by Pereira et al. showed that the nutraceuticals containing low n-6/n-3 in the ratio of 1.4:1 (1.2 g/kg/day nutraceuticals orally by gavage) and high n-9/n-6 in the ratio of 3.7:1 (1.2 g/kg/day nutraceuticals orally by gavage) decreased pain associated with endometriosis compared to the controls, but did not improve fertility in any of the tested groups [86]. Notwithstanding the promising results, all animal studies included in this review (Table 2) need confirmation in human studies.

In conclusion, the association between PUFA intake and the risk of developing endometriosis is unclear (Table 1); however, taking into account the results, women with endometriosis should be recommended to increase n-3 PUFA intake, especially the EPA and DHA, and pay attention to the EPA:AA ratio, which reduce pain-related symptoms (Table 3) and inflammation. A higher intake of PUFAs may be recommended as the perfect element in endometriosis prophylaxis and post-diagnosis of the disease because, in addition to symptom relief, PUFAs play a role in preventing disease pathogenesis.

## 7. *Trans* Fatty Acids (*t*FAs)

Trans-FAs, with one or more double bonds in the trans configuration instead of the usual cis configuration [87], can be found naturally in meat and dairy products as a result of anaerobic bacterial fermentation in ruminant animals. However, most of them have been produced during the industrial processing of vegetable oils during the hydrogenation process [88]. Industrial processing *t*FAs have been mainly categorized as harmful to human health [89]. Food products that usually contain high levels of *t*FAs include cookies, French fries, crackers, doughnuts, margarine, chocolate, and fried chicken [90]. Dietary guidelines recommend a tFA intake below 1% of energy or as low as possible, which helps to prevent diet-related and metabolic diseases in the adult population [42,43,44].

High intake of *t*FAs is associated with higher levels of several inflammatory markers, such as IL-6 and TNF- α [90], which are thought to be involved in the pathogenesis of endometriosis [38]. The essential mediating steps of endometriosis-mediated events may be the activation of inflammatory responses [91]. Thus, the risk of endometriosis is increased through down-regulation of the peroxisome proliferator-activated receptor-γ (PPAR-γ) [92]. PPARγ regulates the expression of adipocyte-derived circulating hormones (adipocytokines) such as adiponectin [93]. Ligands of the PPAR-γ have been described to trigger the reversal of surgically induced endometriosis in rodents [94] and baboons [95].

There are no currently established guidelines for the intake of *t*FAs for women with endometriosis. However, according to current research, it is vital to limit *t*FAs to a minimum or exclude them entirely because tFAs induce pro-inflammatory processes and oxidative stress.

According to meta-analysis results, there is a significant association between tFA intake and the occurrence of endometriosis [45]. Similarly, a prospective study within the Nurses’ Health Study II showed that the intake of *t*FAs was related to increased endometriosis risk. Women in the highest quintile of tFA intake were at a 48% greater risk of endometriosis. In addition, each 1% of energy derived from *t*FAs rather than any other type of fat (e.g., saturated, monounsaturated, polyunsaturated fats) was associated with an increased risk of endometriosis [19]. In contrast, population-based case-control studies have observed inverse associations between endometriosis risk and tFA intake [46].

Nevertheless, from a scientific standpoint, the data is still insufficient. Only a few studies have sufficiently investigated the relationship between *t*FAs and endometriosis. However, based on the data presented here (Table 1), a higher consumption of products containing *t*FAs increases the risk of developing endometriosis. More research is needed to obtain strong evidence of the reduction of *t*FAs with respect to endometriosis risk.

## 8. Summary

**Figure 1:**
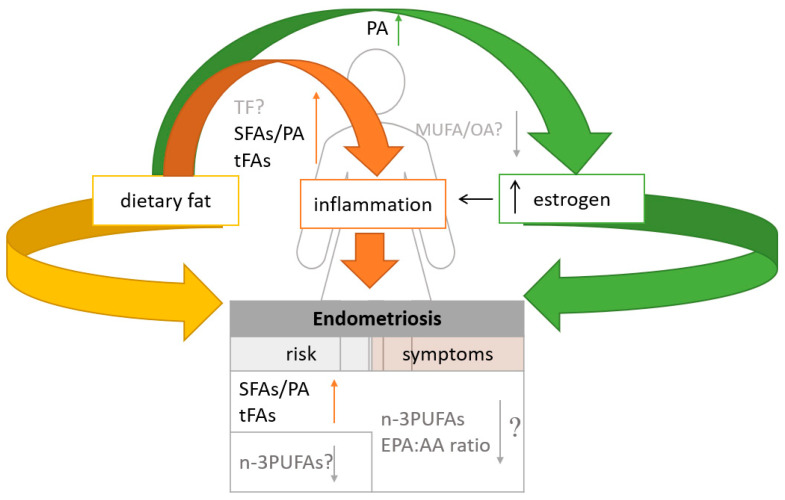
Summary of the influence of dietary fats on endometriosis risk and related symptoms; TF—total fats; SFAs—saturated fatty acids; PA—palmitic acid; *t*FAs—trans-unsaturated fatty acids; MUFAs—monounsaturated fatty acids; OA—oleic acid; n-3 PUFAs—omega-3 polyunsaturated fatty acids; EPA:AA ratio eicosapentaenoic acid to arachidonic acid; ? -doubts; ↓ decrease; ↑ increase.

Endometriosis risk can be controlled and even prevented by modifying dietary fat intake, as dietary fatty acids may contribute to endometriosis by modulating inflammatory pathways and affecting endogenous estrogen production (Figure 1). Inflammation is thought to be one of the major factors in endometriosis. Because inflammatory processes play a major role in the pathogenesis of endometriosis, regulating the amount and types of fat consumed may be recommended for disease treatment. Additionally, Mu et al. observed that women with endometriosis may also have a higher risk of hypercholesterolemia [96], which is why the proper selection of dietary fat sources is crucial in this group. Although there are no clear correlations between total fat intake and endometriosis, the composition of fatty acids in the diet may be linked to the risk of endometriosis [19] and diet therapy. It has been shown that diets rich in saturated fatty acids and/or trans fatty acids may have an adverse effect on the course and treatment of endometriosis. These dietary components stimulate the production of pro-inflammatory cytokines and increase inflammation. In addition, endometriosis is an estrogen-dependent disease, and high consumption of palmitic acid has been proven to increase estrogen production and where elevated levels may be involved in causing inflammation in endometriosis. It was also found that substituting SFAs with MUFAs or n-3 PUFAs eliminates the proinflammatory activity of LPS [56]. MUFAs and n-3 PUFAs decrease proinflammatory cytokine levels and may reduce the risk of endometriosis.

Taking all these results into consideration, dietary recommendations for women at risk or diagnosed with endometriosis should include limiting SFA-rich foods, especially palmitic acid, and trans fatty acids. In terms of unsaturated fatty acids, it may be advisable to increase the consumption of food products containing more polyunsaturated fatty acids, especially oleic acid, and n-3 PUFA, with particular attention to the EPA:AA ratio. They can be particularly important in endometriosis and the reduction of pain symptoms.

Existing uncertain data must be supported by studies offering high comparability, and previous results of experimental studies must be extended. Further well-designed and randomized controlled trials are needed to determine the short-term and long-term efficacy of dietary fat.

## Figures and Tables

**Table 1:** Dietary fat intake and endometriosis.

Study Design	Subject	Results					
		TF	SFAs	MUFAs	PUFAs n-3	PUFAs n-6	*t*FAs
Meta-analysis [45]	The association between i.a. fats and the risk of endometriosis among adult women; 8 publications, including 5 cohorts and 3 case-control.	No effect	↑	No effect	No effect	No effect	↑
Prospective study [19]	The relation between dietary fat intake and the risk of endometriosis; the experimental group n = 1199 women aged 25–42 years with endometriosis; median intake of total fat 24.2–38; SFAs 8.1–13.9; MUFAs 8.9–14.8; PUFAs 4.1–7.0; n-3, 0.4–0.8; n-6, 3.5–6.4; *t*FAs 0.9–2.3 % of energy.	No effect	No effect SFA; ↑ PA	No effect	↓	No effect	↑
Case-control [46]	Dietary risk factors, i.e., fats for endometriosis; the experimental group n = 284 women aged 20–65 years with endometriosis; median intake of total fat 54.8 g, SFAs 19.2 g, MUFAs 20.3 g, *t*FAs 3.2 g.	↓	↓	↓	-	-	↓
Case-control [47]	The relationship between food consumption and nutrient i.a. fat intake with risk of endometriosis; n = 156: n = 78 women with endometriosis and control group n = 78; the regression analyses (odds ratio).	No effect	-	↓ ↓ OA	↓	-	-
Case-control [48]	The relation between *i.a* fats and benign ovarian tumors (BOTs) and nutrients, primarily dietary fat; the experimental group n = 393 women with BOTs, n = 280 women with endometrial tumors; median intake of total fat 48.9 g, SFAs 18.9 g, MUFAs 19.9 g, PUFAs 10.3 g.	↑	↑	↑	↑	↑	-
Case-control [51]	The relation between dietary fat intake and the risk of endometriosis; the experimental group n = 25 women with stage I–IV endometriosis; median intake of SFAs 18.56 g, n-3 PUFAs 0.66 g; n-6 PUFAs 9.61 g.	-	No effect	-	↓	↓	-
In vitro study [36]	The effects of composition of n-3 and n-6 PUFAs on in vitro proliferation of endometrial cells and their production of the cytokine interleukin-8 (IL-8); endometrial cells from women with and without endometriosis.	-	-	-	↓	-	-

↓ decrease of endometriosis risk; ↑ increase of endometriosis risk.

**Table 2:** PUFAs intake and endometriosis risk (animal study).

Subject	Results	
	n-3 PUFAs	n-6 PUFAs
Dietary supplementation with fish oil containing EPA/DHA (experimental group) or olive oil (control group)—effect on surgically induced endometriosis in rabbit n = 38 [82].	↓	-
The effect of n-3 PUFAs in a mouse endometriosis model by making full use of two types of genetically modified mice: fat-1 and 12/15-LOX KO [74].	↓	-
Dietary fish oil supplementation and formation of endometriosis-associated adhesions in a chimeric mouse model; standard or menhaden fish oil (~40% omega-3 fatty acids)—supplemented diets for ≥2 weeks before initiation of experimental endometriosis [84].	↓	-
The anti-inflammatory effect of EPA (n-3) compared with LA (n-6) in an endometriosis rat model [41].	↓	-
PUFA n-3 and the establishment of endometriosis-like lesions; Wild Type (WT) and transgenic Fat-1 mice (high levels of endogenous n-3) endometriosis model; systemic host n-3 levels impact anti-inflammatory during early establishment of endometriosis [85].	↓	-
The nutraceuticals n-6/3 and n-9/6 effect on endometriosis-associated infertility and pain; rats; fertility groups: control with endometriosis omega-6/3 (1.2 g/kg/day); omega-9/6 (1.2 g/kg/day); “pain groups”: control with endometriosis omega-6/3 (1.2 g/kg/day); omega-9/6 (1.2 g/kg/day) [86].	↓	↓

↓ decrease of endometriosis risk or progression.

**Table 3:** PUFA intake and pain-related symptoms in endometriosis.

Study Design	Subject	Results	
		PUFAs n-3	PUFAs n-6
Comprehensive review [77]	Diet and dysmenorrhea among women with endometriosis; 11 trials with different designs, including a total of 1433 women.	↓	-
Double-blind, randomized, placebo-controlled trial [78]	Supplementation with i.a. PUFA n-3 (1000 mg fish oil), and pain related symptoms in women with endometriosis; n = 20, aged 12–25 years, 6 month.	↓	-
Cohort-study [79]	Dietary supplementation: 30 compositions containing i.a. linoleic acid (n-6), α-linolenic acid (n-3) n = 90 women with endometriosis; 3 months.	↓	↓

↓ decrease pain-related symptoms.

## Data Availability

Not applicable.
